# Vectorizing the spatial structure of high-harmonic radiation from gas

**DOI:** 10.1038/s41467-019-10014-5

**Published:** 2019-05-01

**Authors:** F. Kong, C. Zhang, H. Larocque, Z. Li, F. Bouchard, D. H. Ko, G. G. Brown, A. Korobenko, T. J. Hammond, Robert W. Boyd, E. Karimi, P. B. Corkum

**Affiliations:** 1 Department of Physics, University of Ottawa, 25 Templeton St., Ottawa, ON K1N 6N5 Canada; 2 Joint Attosecond Science Laboratory, University of Ottawa and National Research Council of Canada, 100 Sussex Drive, Ottawa, K1A 0R6 Canada; 3 The Institute of Optics, University of Rochester, Rochester, NY 14627 USA; 4 Department of Physics, Institute for Advanced Studies in Basic Sciences, Zanjan, 45137-66731 Iran

**Keywords:** High-harmonic generation, Nonlinear optics

## Abstract

Strong field laser physics has primarily been concerned with controlling beams in time while keeping their spatial profiles invariant. In the case of high harmonic generation, the harmonic beam is the result of the coherent superposition of atomic dipole emissions. Therefore, fundamental beams can be tailored in space, and their spatial characteristics will be imparted onto the harmonics. Here we produce high harmonics using a space-varying polarized fundamental laser beam, which we refer to as a vector beam. By exploiting the natural evolution of a vector beam as it propagates, we convert the fundamental beam into high harmonic radiation at its focus where the polarization is primarily linear. This evolution results in circularly polarized high harmonics in the far field. Such beams will be important for ultrafast probing of magnetic materials.

## Abstract (web summary)

Ultrafast pulses with controlled parameters are desirable in many applications including probing materials and their interaction with light. Here the authors demonstrate a technique for polarization control of XUV beam using high-harmonic generation and polarization shaping.

## Introduction

Recent advances allow us to shape the spatial mode of a laser beam in phase and polarization at will, point by point, in their transverse plane^1^. Such techniques commonly rely on devices that are composed of aligned liquid crystal molecules whose anisotropy can be tuned with electric fields^2^. Despite their utility in the visible and near infrared, such devices cannot be used in the extreme ultraviolet (XUV). However, it is possible through high harmonic generation to upconvert the structured fundamental beams to the extreme ultraviolet^3–8^.

Nevertheless, it is much more common to shape the fundamental beam that produces high harmonics in time rather than in space. A typical method for producing isolated attosecond pulses uses a fundamental pulse with time varying polarization^9–11^. This method relies on the fact that conversion from the fundamental to the XUV requires nearly linear polarization^12^. Generating circularly polarized XUV beams by mixing counter rotating bicircular field with different colors is another good example of shaping the laser field in the temporal domain. The field is shaped to have three-fold symmetry and enables the tunneled electron to recombine with its parental ion every one third of the fundamental optical cycle^13–15^. The field shaping in temporal domain enables efficient generation of circularly polarized high-order harmonics. Alternatively, the circularly polarized harmonics are also reported by mixing two non-collinear bicircular driving laser beams^16,17^. Reducing the relative angle is helpful to optimize the phase matching in producing XUV radiation. To further exploit the spatial degree of freedom, the beam-mixing scheme evolves into the direct modulation of spatial modes of laser beams.

In this experiment, we use liquid crystal technology^18,19^ to shape the polarization of a beam in space rather than in time, which we refer to as a vector beam^20^. Such a beam naturally evolves upon propagation, with the polarization structure of the beam changing from near to far field^21,22^. By exploiting the natural evolution of the vector beam, we convert the fundamental beam into high harmonic radiation at its focus where its polarization is primarily linear. This evolution of the generated high harmonic beam results in circularly polarized XUV radiation in the far field. Comparing to the non-colinear beam-mixing schemes, the laser beam can be modulated pixel-by-pixel enabling a complete and more flexible control of its spatial properties. This opens a field of vectorizing the radiation in both strong infrared and XUV radiation.

## Results

### Shaping the polarization of the fundamental beam

**Fig. 1:**
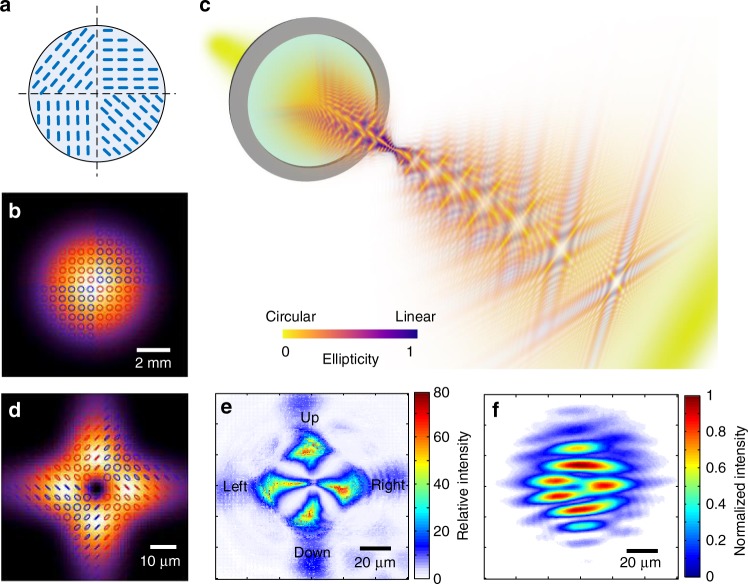
Shaping and characterizing the optical polarization of the fundamental driving laser beam. **a** Design of the liquid crystal phase plate. The short blue lines represent the orientation of the aligned liquid crystal molecules. **b** Intensity and polarization plot of the fundamental beam in the near field; the blue and red parts distinguish four segments delayed by *π*/2-phase. **c** The evolution of polarization state of a vector beam while it is being focused. The yellow-colored part shows where the polarization is circular and blue-colored part shows where it is linear. The linear parts start to show up at the interface between adjacent segments and are dominant at the focal plane. The circular state is restored when the beam propagates away from the focal plane. **d** Intensity and polarization plot of the driving infrared beam at the focal plane. Linear polarization states are constructed within the four intense lobes, where high harmonics are produced. **e** Measured ratios between the maximum and minimum intensity at each point in space for various rotation angles of the linear polarizer. A higher distinguish ratio indicates that the local polarization state is more linear. **f** The interference pattern between the fundamental vector beam and Gaussian beam at the focal plane. The shifted fringe pattern indicates a *π*-phase difference between the opposing segments, and each segment is phase delayed from this neighbor by *π*/2

The liquid crystal plate that we use to shape the driving laser beam is tuned^19,23^ to give half-wave retardation at the fundamental wavelength. The liquid crystal molecules are aligned in different orientations in each quadrant, as shown in Fig. 1a. With this device and a quarter-wave plate, we can generate the beam shown in Fig. 1b, where the circles indicate circularly polarized light and the red (blue) color indicates the opposite handedness. Specifically, we modify the transverse profile of a Gaussian fundamental beam such that its optical properties vary from one quadrant to another. In this case, adjacent quadrants are defined by opposite circular polarizations with different optical phases. In the far field, we show that this beam evolves into one with linear polarized light in each of four quadrants with neighboring segment perpendicularly polarized and each phase delayed by *π*/2. This beam then interacts with a gas-phase nonlinear medium, thus creating high harmonics in the XUV where the spatial structure of the original linearly polarized regions is preserved. As with the fundamental, the polarization state of the resulting XUV vector beam will also evolve as the beam propagates from near to far field (see Supplementary Figs. 1, 2). We show that in the far field, this beam is composed of circularly polarized light in each quadrant, with adjacent quadrants having different handedness.

We focus the infrared beam with a 30 cm lens into a pulsed Argon gas jet placed within a vacuum chamber. The diffractive nature of light causes the field from each segment to interfere and form new local polarization states at the focal plane. This conversion is illustrated in Fig. 1c, where we see how ellipticity evolves while the driving beam is focused: the incident beam is entirely circularly polarized, a feature represented by its yellow color. After being focused by a lens, the polarization approaches to a linear polarization state, represented by the purple sections of the beam. The linear polarization appears first at the interface between two adjacent segments defined by counter rotating circular states. The orientation of the linear polarization is determined by the quadrants’ relative phase difference. As the beam propagates diffraction increases the overlap between two adjacent quadrants. At the focus, Fig. 1d, the fundamental beam is linearly polarized where the field is most intense, and these regions are where high harmonics will be generated.

### Producing high-harmonic radiation with vector beams

Efficient high-harmonic production relies on linearly polarized driving laser fields^12,24^. The local ellipticity of the fundamental beam at the focal plane can be obtained by measuring the transmitted intensity profile through a linear polarizer. By rotating the linear polarizer, the light field is projected onto different linear bases. The ratio between the minimum and maximum intensity of each point in space is plotted in Fig. 1e. The strong modulation confirms that the field propagation constructively interferes to create locally linearly polarized light at the focus, thus enabling efficient high harmonic generation. The relative phase delay is measured by interfering the fundamental beam with a diagonally polarized plane wave. The resulting interference pattern is shown in Fig. 1f. The shifted fringe pattern indicates a *π*-phase difference between the opposing segments, and each segment is phase delayed from its neighbor by *π*/2.

Next, we analyze what we expect as the beam is converted to harmonics. Between the adjacent segments, the driving field has a *π*/2 phase difference. This phase difference between the quadrants is transferred to XUV dipole emission by multiplying them by *N* *=* 2*k* + 1, where *N* is the high harmonic order and *k* is an integer. Then the XUV emitting phase *φ*_XUV_ of the four segments becomes^25^:

1 $${\varphi _{{\mathrm{XUV}}} = N\left[ {\begin{array}{*{20}{c}} {\frac{\pi }{2}} & 0 \\ \pi & {\frac{{3\pi }}{2}} \end{array}} \right] = \left( {2k + 1} \right)\left[ {\begin{array}{*{20}{c}} {\frac{\pi }{2}} & 0 \\ \pi & {\frac{{3\pi }}{2}} \end{array}} \right] = \left\{ {\begin{array}{*{20}{c}} {\left[ {\begin{array}{*{20}{c}} {\frac{{3\pi }}{2}} & 0 \\ \pi & {\frac{\pi }{2}} \end{array}} \right]\;{\mathrm{mod}}\;2\pi ,\;{\mathrm{for}}\;k\;{\mathrm{odd}}} \\ {\left[ {\begin{array}{*{20}{c}} {\frac{\pi }{2}} & 0 \\ \pi & {\frac{{3\pi }}{2}} \end{array}} \right]\;{\mathrm{mod}}\;2\pi ,\;{\mathrm{for}}\;k\;{\mathrm{even}}.} \end{array}} \right.}$$

Equation 1 shows that the phase pattern is preserved even after the frequency up-conversion of all odd order harmonics. After the high harmonics are generated at the focus, the XUV radiation propagates in free space. Since both the local polarization and the *π*/2 phase retardation between neighboring quadrants are preserved on conversion, the XUV beam will restore the circular polarization when it propagates away from the generating site. The intensity induced dipole phase will also contribute to the phase structure in each quadrant. However, each quadrant has same intensity distribution and therefore the same dipole phase structure in the emitting phase. Therefore, the phase relation between the quadrants described in Eq. (1) is still valid. The up-converted phase has a reversed handedness in the helical phase fronts between adjacent harmonic orders. As a result, the harmonic emission of adjacent harmonic orders will have opposite handedness in the same segment, since the phase differences between the orthogonal linear components are *π*/2 and −*π*/2, respectively.

### Characterizing high harmonic beams with complex polarizations

**Fig. 2:**
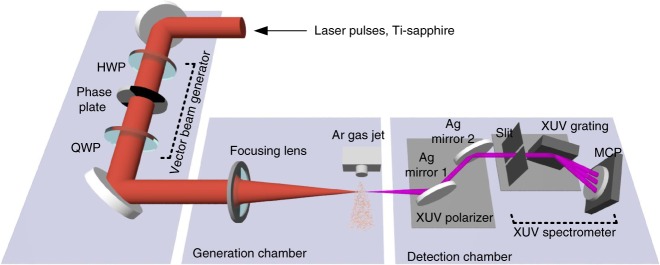
Experimental setup for measuring XUV polarization using an XUV polarizer. The linearly polarized driving laser beam is prepared by going through a half-wave plate, a designed phase plate shown in Fig. 1a and a quarter-wave plate to convert into circular state. It is then focused by a 30 cm lens into a vacuum chamber to interact with argon noble gas. The generated XUV radiation propagates through a XUV linear polarizer placed 50 cm from the gas jet and in the far field for the XUV light. The reflectivity of a silver mirror in the XUV is polarization dependent. With a 23-degree angle of incidence to a pair of silver mirrors we construct a XUV polarizer in front of a XUV spectrometer to select one linearly polarized state

**Fig. 3:**
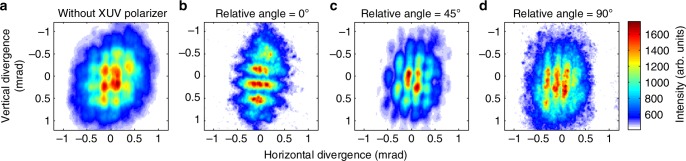
Reconstructed two-dimensional beam intensity profiles of the 25th harmonic beam. The spectrally resolved 2D profile of generated XUV beams can be reconstructed by translating the spectrometer while recording the spectrogram. **a** Measured beam profile without going through the XUV polarizer. **b**–**d** Projected intensity profiles for different linear bases. By changing the relative angle **b** 0°, **c** 45°, **d** 90° between the vector beam generator and the XUV polarizer, we measure the intensity distribution of 25th harmonic beam after going through the XUV polarizer. Driven by the designed vector fundamental beam, the generated XUV radiation consists of both s-polarized and p-polarized components shown in **b**, **d**. The intensity does not vanish at bright spots, while changing the relative angle between the polarizer and polarization of the beam. **c** When the relative angle is set to 45°, modulation appears in both vertical and horizontal directions

**Fig. 4:**
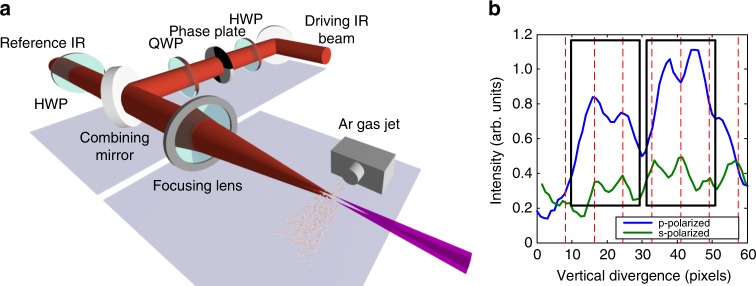
Measuring interference between an XUV vector beam and a XUV reference beam. **a** Experimental setup. The polarization of the reference beam is controlled by a half-wave plate (HWP) for the fundamental. The XUV beams are spectrally resolved and spatially overlapped in an XUV spectrometer. The interference images are recorded by the micro-channel plate (MCP). **b** Interference between the XUV reference and vector beam. The blue curve shows the fringes when the reference beam is p-polarized, and the green curve shows the fringes when the reference beam is s-polarized. The dotted red lines indicate periods of the modulation. All the red dotted lines aligned with all the peaks of the green curve, which indicates the s-component at intersections are all in phase. For the p-component shown with the blue curve, either the peaks or the valleys are aligned with the red dotted line, and that indicates p-components at intersections that are out of phase with respect to the adjacent one

We characterize the polarization distribution of the XUV beams in space by measuring their two-dimensional spatial profiles in the far field. As illustrated in Fig. 2, to distinguish the two orthogonal linear polarization states, we implement two parallel silver mirrors as an XUV polarizer^26^ with ~1:10 extinction ratio (see Supplementary Fig. 3). First, the intensity beam profile of the 25th harmonic, Fig. 3a, is reconstructed without passing through the XUV polarizer. The spectrally-resolved two-dimensional spatial profile is obtained by horizontally translating the XUV spectrometer across the XUV beam (see Methods section). The beam consists of an array of bright spots, which is predicted to be circularly polarized. The multiple-spot distribution is the two-dimensional Fourier transform of harmonics distribution at the focal plane. It is also a signature of the high-order nonlinearity of this process, in which the high harmonics are produced nonlinearly as a function of the intensity and ellipticity. Such nonlinear transfer function is also Fourier transformed and convolutes in the far field, which results in multiple spots structure in the detecting plane. Furthermore, the adjacent spots have the opposite handedness, which they inherit from the driving field. We prove this in Fig. 4 by measuring the relative phase in the linear basis.

Using the same approach, we reconstruct the intensity profile of the transmitted beam, after the XUV vector beam passes through the XUV polarizer. In lieu of rotating the XUV polarizer, we rotate the polarization of the incident beam by introducing a half-wave plate in front of the liquid crystal plate in the experimental setup (see Methods section for further details). If the polarizer is set at a 0° relative angle, with respect to the polarization direction of the upper and lower segments of the fundamental as labeled in Fig. 1e, the transmitted beam shows horizontal fringes (Fig. 3b). These fringes can be interpreted as two-source interference of the two generating segments (upper and lower ones), which are displaced in the vertical direction. Each dark node in the vertical direction indicates a *π*-phase jump in the XUV due to the interference. Radiation from the other two segments (left/right) is not transmitted, since it is orthogonal to the direction of the XUV polarizer, which is aligned with the polarization of the upper/lower segments.

If we change the waveplate angle, the intensity will never vanish at the position of bright spots shown in Fig. 3a. This shows the XUV field has nonzero component in every direction and is consistent with the behavior of a circular/elliptical polarization state. Figure 3c shows the intensity profile when the polarizer is oriented at 45°. The intensity profile has a similar multi-spot structure to that in Fig. 3a, and all segments transmit through the XUV polarizer. Similar behavior is also observed in other harmonic orders (see Supplementary Fig. 4). The interferometric modulations occur in both vertical and horizontal directions, since radiation from all four generating segments can be equally transmitted through the XUV polarizer. This intensity profile also disproves the linear polarization at those segments, since adjacent segments would have orthogonal −45° polarization and would not transmit through the 45° XUV polarizer.

If the relative angle reaches to 90°, the transmitted intensity shows vertical fringes, as in Fig. 3d, which is similar to the 0° case but in an orthogonal direction. These fringes come from the interference between the left/right segments, which are horizontally displaced in space. The signal-to-noise ratio is degraded in these measurements due to the scattering and low reflectance of the silver mirrors in the XUV polarizer. Figure 3b, d depict the intensity distribution when the vector XUV beam is projected to the orthogonal linear basis. This again illustrates, at the intersection of these two sets of fringes, the bright spot of the XUV beam has both orthogonally polarized linear components.

Next, we turn to the phase relation in the XUV vector beam and confirm experimentally the opposite handedness of adjacent bright spots. For our measurement, we employ a linear reference XUV beam, produced by a separated and more tightly focused high-harmonic source. This reference XUV beam interferes with the vector XUV beam we generate, shown in Fig. 4a, over its whole transverse profile. We can control the polarization of the reference XUV beam with a half-wave plate acting on its fundamental, since the generated XUV beam has the same linearly polarized state as its driving field.

In the relative phase measurement, we fix the position of the XUV spectrometer and allow only two vertically-displaced adjacent XUV segments to pass through the spectrometer slit. They are identified by the two boxes in Fig. 4b. Then we use the linear reference XUV beam to interfere with the two transmitted segments. The blue and green curves in Fig. 4b show the interferences when the reference XUV beam is p- and s-polarized, respectively. If the two segments have the same handedness, then the modulation in each segment will line up for both polarizations. If they have opposite handedness, then there will be a phase shift of one cycle with respect to the other. In the figure, we see that the intensity is modulated vertically as expected. The dashed red lines in Fig. 4b reference the peaks of modulation periodicity.

When the reference beam is s-polarized (green curve), all peaks are aligned with the reference lines in both left and right boxes. In the case of p-polarized interference, the peaks are in-phase with the dashed reference lines in the left box and out-of-phase in the right box. This shows that the p-polarized components have a *π*-phase jump between the adjacent spots. The in-phase s-component and out-of-phase p-component cause counter rotating polarization between the two segments. With this measurement, we can perform phase sensitive optical tomography on the vector XUV beam along two orthogonal polarization bases.

## Discussion

The ability to control the spatial mode of the driving beam enables us to generate more complex beams by engineering their transverse profile. The phase retardation can be designed by patterning different phase structures on the liquid crystal plate, while the polarization of XUV can be changed by changing the driving polarization. In this case, the high harmonic generation process provides access to transferring the spatial manipulation to XUV beams. Such manipulation is not only limited to the 2D transverse plane but can be extended to 3D space while the beam is propagating and transforming. The generation of complex polarization states will enable increasingly capable table-top XUV sources to probe the dynamics of polarization sensitive systems^27^.

One important application of circularly polarized XUV is the sensitivity of magnetic domains or chiral molecules to the handedness of light. Currently, both the structure and dynamics of magnetic domains are measured with circularly polarized harmonics created by manipulating the sub-cycle time structure of the fundamental beam. Specifically, these experiments use a driving beam composed of counter rotating light at the fundamental frequency and its second harmonic^15^.

Using the spatial structure of the fundamental rather than the time structure has potential advantages. The polarization remains linear in the region where intensity is highest in our approach. This results in a higher fraction of the energy that can produce high harmonics relative to polarization gating in temporal domain (see Supplementary Note 1 and 2). In addition, the monochromatic driving field leads to a higher cut-off energy, which is mainly limited by the damage threshold of the liquid crystal plate, as required by the 3*U*_p_ cut-off law^12–14,28^ under a same ionization rate. Expanding the beam size on the liquid crystal plate or deploying water cooling system will increase the damage threshold. The extended cut-off that the vector beam approach appears to offer will be important for high photon-energy probes. Structuring the spatial mode of single laser beam provides a paraxial geometry that generally favors phase matching. The interaction length will be limited by the variation of the polarization state and accumulated phase during propagation if the thickness of the interaction medium is significantly increased compared to the Rayleigh length.

High harmonic vector beams such as these are ideal for probing circular dichroism^27,29^ as they can simultaneously provide counter rotating circular polarization states at XUV wavelengths. The spatial separation of circularly polarized spots permits the isolation of one particular state by spatial filtering. Together with the inherent temporal resolution of high harmonic sources, this technique may be used to study how magnetic domains can be switched^30^. This result also proves the capability of using the spatial properties of vector driving beams to generate high harmonics with novel complex spatial profiles. That is, it leads to another degree of freedom which we can design and engineer for table top harmonic based XUV sources.

## Methods

### Obtaining spectrally-resolved two-dimensional intensity profile

Since our vector beam does not have azimuthal symmetry in either its intensity or polarization state, a two-dimensional beam profile measurement is required for every harmonic order. We send the XUV beam to an XUV spectrometer to get spectrally resolved signals. Any variation of the beam intensity profile in the vertical direction is directly recorded on our spectrometer in a single image. Then we take multiple images while translating the XUV spectrometer horizontally to obtain the intensity variation along horizontal direction. The two-dimensional image of the XUV beam profile is reconstructed by stacking the translated recorded spectrograms according to the position of the XUV spectrometer. We perform this beam profile measurement both with and without the XUV polarizer.

### Projecting the vector XUV beams onto different linear polarization states

The polarizer is difficult to rotate physically. In addition, it is difficult to align with the beam propagation direction, so that the incident angle can be maintained while the XUV polarizer is rotated. Instead, we change the polarizing orientation of the incident driving laser beam, which is equivalent to physically rotating the XUV polarizer, except for the polarization selectivity of the XUV grating. In our setup, two parallel mirrors are installed in front of the spectrometer to ensure the same incident angle. To rotate the polarization state of the incident fundamental driving laser beam, a half-wave plate is placed in front of the phase plate. When the half-wave plate is rotated by a small angle *θ*, the polarization of the incident beam is rotated by 2*θ*. After going through a phase plate, the polarization of each segment is rotated by −4*θ* with respect to the phase plate and obtains a −2*θ* rotation in the lab frame. Then the quarter-wave plate is rotated by −2*θ*, to retain the same incident angle between optical axes of the wave plate with the incident polarization.

## Supplementary information

Supplementary Information

## Data Availability

The data that support the findings of this study are available from the corresponding author on reasonable request.
