# Supplementary material for: Vectorizing the spatial structure of high-harmonic radiation from gas
Source: Nat Commun. 2019 May 1;10:2020. doi: 10.1038/s41467-019-10014-5 (PMC6494894; doi:10.1038/s41467-019-10014-5)
Supplement: Supplementary file 1 — Supplementary Information [file 41467_2019_10014_MOESM1_ESM.pdf]

Supplementary Information

Vectorizing the spatial structure of high-harmonic radiation from gas

Kong *et al.*

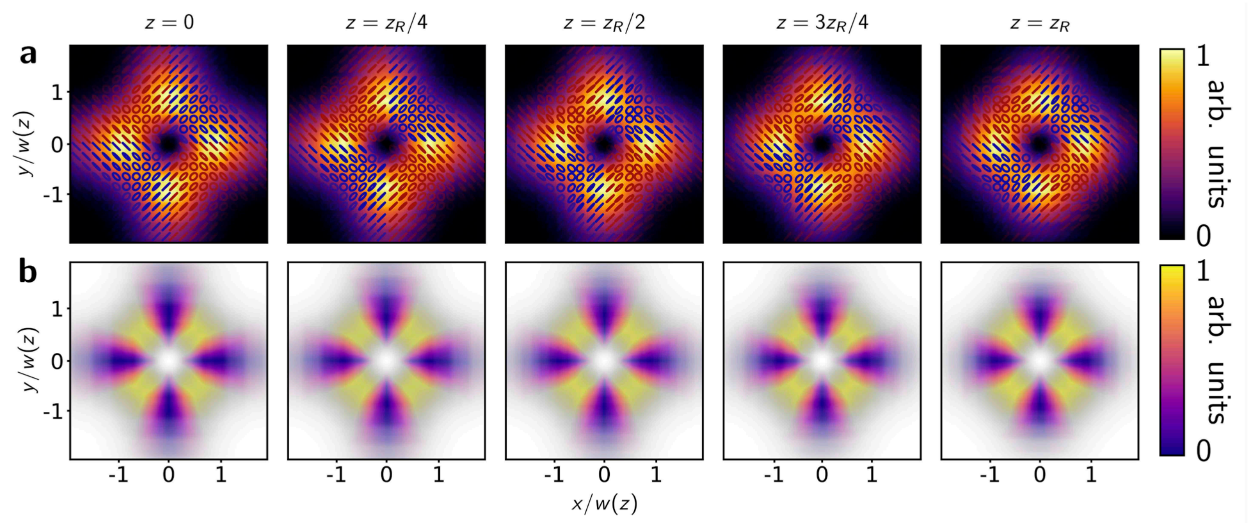

Supplementary Figure 1 Profile of the focused structured pump beam The beam profile is considered along one Rayleigh range from its focus. **a** Total intensity of the beam overlaid with its space-varying polarization pattern. Left-handed polarized ellipses are drawn in red while right-handed polarized ellipses are drawn in blue. **b** Absolute value of the third Stokes parameter indicating the degree to which the beam is elliptical, where 0 corresponds to purely linear polarization and 1 corresponds to purely circular polarization.

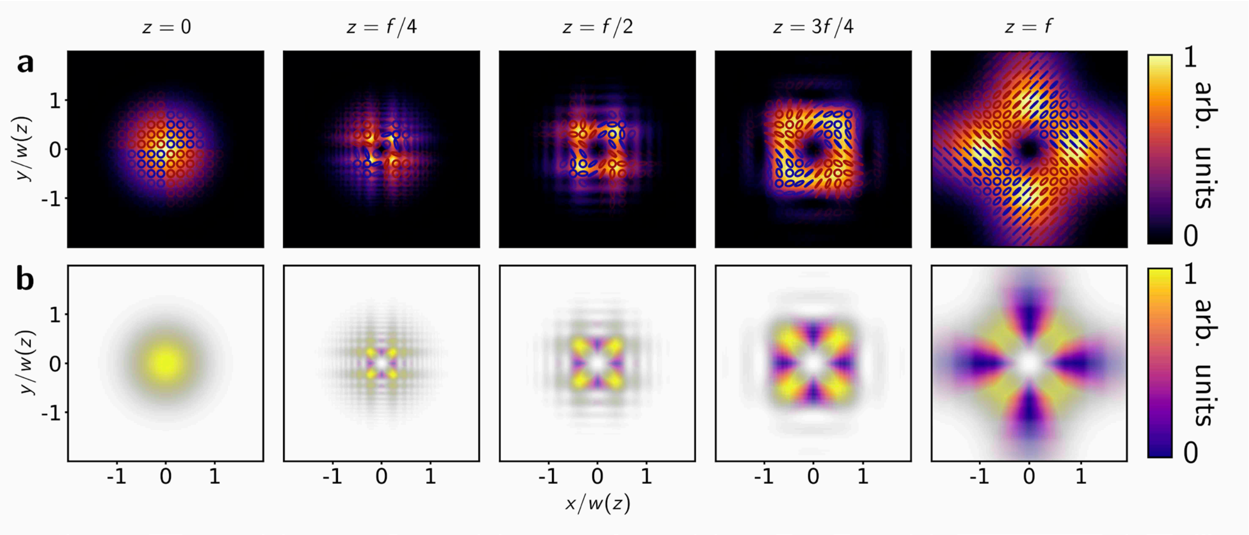

Supplementary Figure 2 Profile of the structured pump beam upon being focused The beam profile is considered after the phase plate to its focus, where f is the focal length. **a,** Total intensity of the beam overlaid with its space-varying polarization pattern. Left-handed polarized ellipses are drawn in red while right-handed polarized ellipses are drawn in blue. **b**, Absolute value of the third Stokes parameter indicating the degree to which the beam is elliptical, where 0 corresponds to purely linear polarization and 1 corresponds to purely circular polarization.

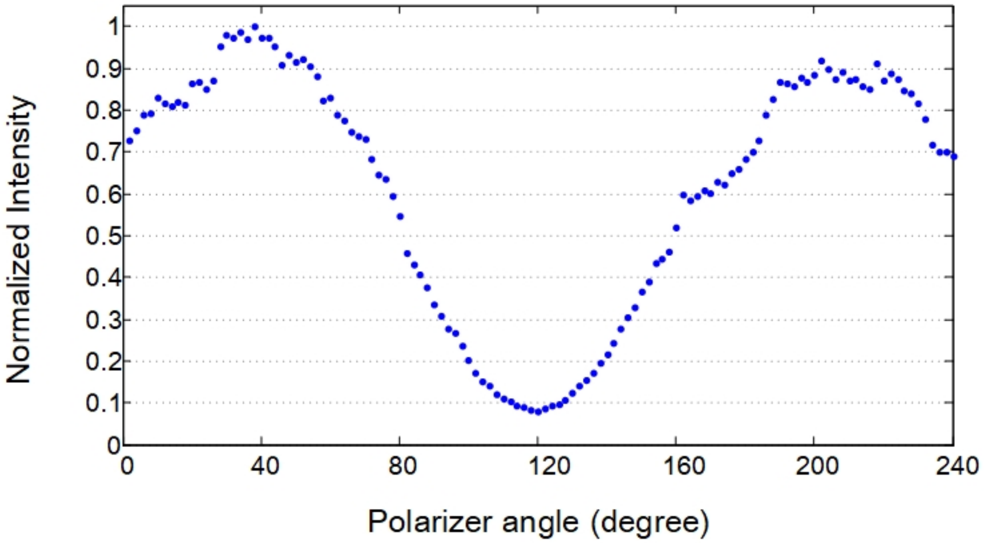

Supplementary Figure 3 Linear calibration of XUV spectrometer Normalized transmitted intensity of XUV beam at the 25th harmonics order versus different equivalent polarizer angles, with respect to the direction of the polarization of the XUV beam. The extinction ratio between s- and p-polarizations is ~10:1.

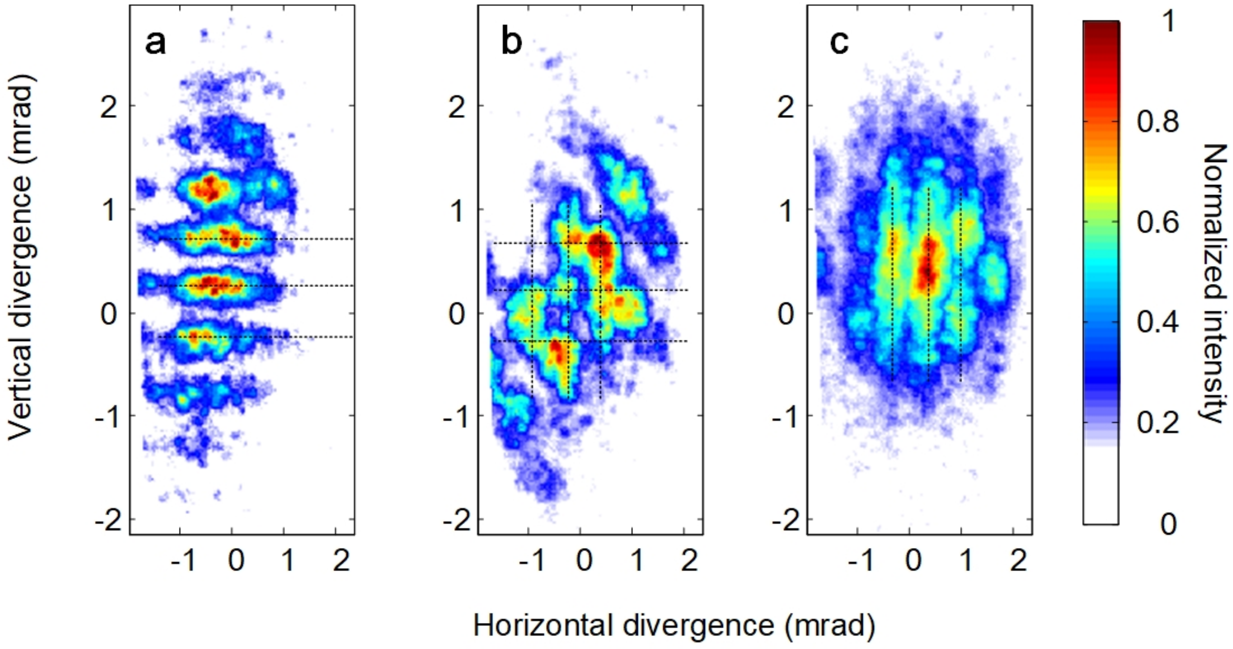

Supplementary Figure 4 Reconstructed two-dimensional beam intensity profiles of the 21st harmonic beam The spectrally resolved 2D profile of generated XUV beams can be reconstructed by translating the spectrometer while recording the spectrogram. **a-c**, Projected intensity profiles for different linear bases. By changing the relative angle **a** 0°, **b** 45°, **c** 90° between the vector beam generator and the XUV polarizer, we measure the intensity distribution of 21st harmonic beam after going through the XUV polarizer. Driven by the designed vector fundamental beam, the generated XUV radiation consists of both s- and p-polarized components shown in **a** and **c**. The intensity does not vanish at bright spots, while changing the relative angle between the polarizer and polarization of the beam. **b**, When the relative angle is set to 45°, modulation appears in both vertical and horizontal directions.

Supplementary Note 1 Comparison of temporal and spatial polarization gating

We determined the fraction of the energy that can produce high harmonics by turning on and off the ellipticity dependence in the semi-classical simulation.

(1) We assumed that the intensity remained the same, but the polarization was linear, and we calculated the single atom conversion efficiency.

(2) Then we used the actual vector beam polarization profile and performed the same calculation.

The excursion of the electron is calculated by classical trajectory in the presence of the laser field. The spread of the wave packet is estimated by its perpendicular velocity[1].

Then the yield is calculated point by point as a function of local intensity and helicity at the focal plane. The laser parameters are 50 fs pulse, 3×10^{14} W cm^{-2} peak intensity interacting with argon gas. Supplementary Fig. 5a shows the 25th harmonic yield when the local fields are artificially turned into linearly polarized. Supplementary Fig. 5b shows when the helicity is included. Harmonics are only generated from the four lobes where the polarization is near linear (and the intensity is strongest). No harmonics are produced where the polarization is elliptical or circular (and the intensity is low).

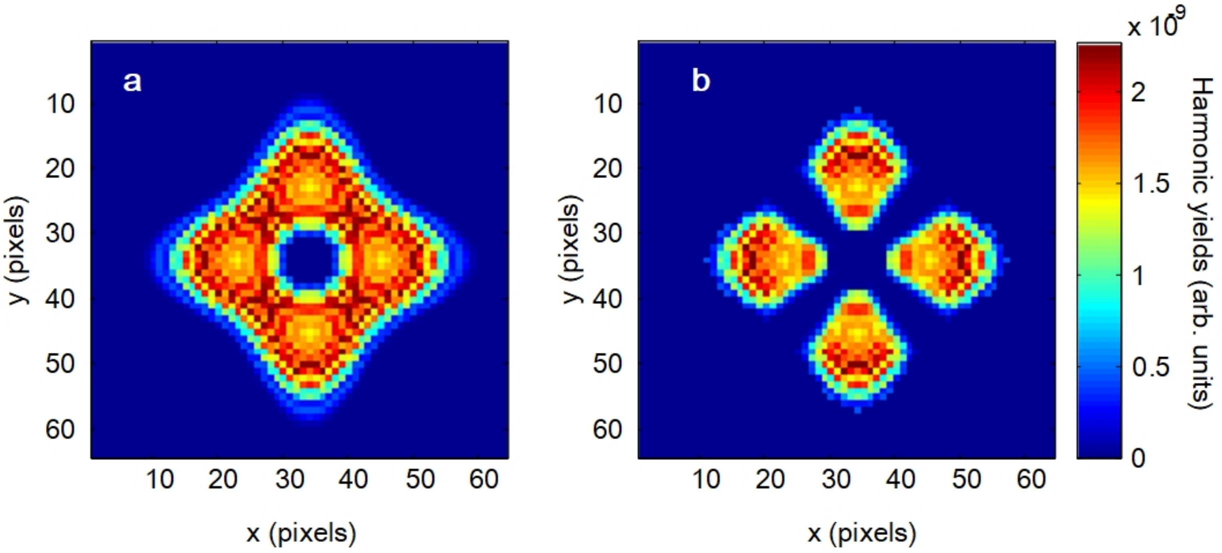

Supplementary Figure 5 25th harmonic yields in the near field a the 25th harmonic yield in the near field when the field is all linear. **b** the 25th harmonic yield in the near field after the polarization shaping. The harmonics are only produced where the field is high, and polarization is closed to linear.

Supplementary Note 2 The fraction of the energy that can produce high harmonics of spatial polarization shaping

With respect to the fraction of the energy that can produce high harmonics for different orders due to polarization shaping, we divide the total high harmonic yield in the right Supplementary Fig. 5b by that in the Supplementary Fig. 5a for each different order. Then the ratio verses the harmonic order is plotted in Supplementary Fig. 6. We can see that, as the order goes higher, the yield is more sensitive to the ellipticity and therefore the fraction of the energy that can produce high harmonics is lower.

This approach can be considered as a spatial analogy to the temporal domain polarization gating. While temporal polarization gating works, a fraction of the laser energy is wasted since there is no HHG when the polarization is not nearly linear. In the temporal polarization gating, more than half of the energy is wasted, since the main peak of the electric field is elliptically polarized and only half cycle field (even not the strongest one) is used to produce high harmonics. In the spatial case, the flexibility of designing the phase pattern ensures that most energy goes into linearly polarized regions. Supplementary Fig. 6 illustrates a promising efficiency that more than half of the energy is used to produce high harmonics in the spatial polarization gating.

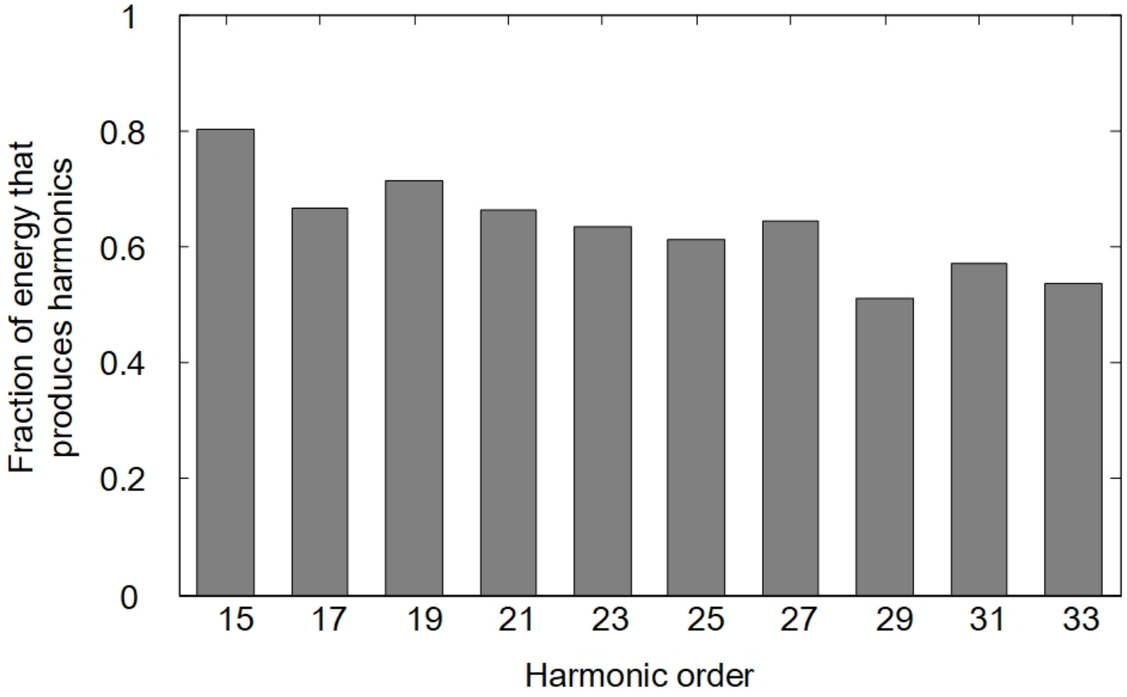

Supplementary Figure 6 The fractions of the energy that can produce high harmonics of different harmonic orders Comparing to the linearly polarized driving laser field, the fraction of the energy that can produce high harmonics gradually reduces as the order of harmonics goes higher.

Supplementary Reference

1. Ivanov, M. Y., Spanner, M. & Smirnova, O. Anatomy of strong field ionization. *J. Mod. Opt.* **52,** 165–184 (2005).
